# A Multicenter Retrospective Study of Cystic and Alveolar Echinococcosis Patients in the United States (1997–2024)

**DOI:** 10.4269/ajtmh.25-0453

**Published:** 2026-02-03

**Authors:** Treana Mayer, Mary Ellen Krienke, Daniel B. Chastain, Christine M. Budke, Nelson I. Agudelo Higuita, Susan VandeWoude, Andrés F. Henao-Martínez

**Affiliations:** ^1^ Department of Microbiology, Immunology, & Pathology, College of Veterinary Medicine & Biomedical Sciences, Colorado State University, Fort Collins, Colorado;; ^2^ Department of Internal Medicine, Anschutz Medical Center, University of Colorado, Aurora, Colorado;; ^3^ Department of Clinical and Administrative Pharmacy, University of Georgia College of Pharmacy, Albany, Georgia;; ^4^ Department of Veterinary Integrative Biosciences, Texas A&M University, College Station, Texas;; ^5^ University of Oklahoma Health Campus, Oklahoma City, Oklahoma;; ^6^ Instituto de Enfermedades Infecciosas y Parasitologia Antonio Vidal, Tegucigalpa, Honduras

## Abstract

Echinococcoses are neglected zoonotic diseases caused by larval cestodes of the genus *Echinococcus*. Regions of the United States rarely experience locally acquired infections, and most contemporary cases are presumed to be imported, predominantly from hyperendemic regions such as Central and East Asia, the Middle East, and North Africa. However, significant knowledge gaps remain regarding the current disease burden within the US healthcare system. The epidemiology, baseline clinical features, and outcomes are summarized for patients diagnosed with cystic echinococcosis (CE) or alveolar echinococcosis (AE) in the present study using retrospective, deidentified data from the TriNetX research network, a federated database that encompasses 89 healthcare organizations and more than 126 million patients (1997–2024). Individuals with *Echinococcus* infection were identified using *International Classification of Diseases, 10th Revision*, code B67, and demographic characteristics, geographic distribution, medical and surgical interventions, and mortality were assessed from 2003 to 2023. More than 36,000 patients with any B67 diagnosis were identified, of whom 728 had codes specific to CE, and 75 had codes specific to AE. There were more CE cases in the Southern United States, whereas most AE were reported in the Northeastern United States. Many patients were asymptomatic, with limited use of diagnostic imaging or serologic testing. Antiparasitic medication or surgical procedures were recorded most commonly in AE patients (18%), with mortality rates between 28% and 38% at 20 years post-index. Although locally acquired AE and CE appear to be emerging in the Northeastern United States, the overall prevalence remains low nationwide. The authors advocate for heightened awareness of echinococcoses among US-based clinicians and recommend prospective surveillance studies to improve clinical outcomes and understanding of local transmission risks.

## INTRODUCTION

Echinococcoses are neglected zoonotic parasitic infections caused by the larval stage of different cestode species belonging to the genus *Echinococcus*. From a public health and medical perspective, the most important species are *Echinococcus granulosus sensu lato* (*E. granulosus sensu lato*) complex and *Echinococcus multilocularis* (*E. multilocularis*), which are the causative agents of cystic echinococcosis (CE) and alveolar echinococcosis (AE), respectively. The adult tapeworm resides in the small intestine of its definitive carnivore mammalian host, shedding infectious ova in its feces. After ingestion of an egg by an intermediate host, a fluid-filled metacestode cyst can develop in any organ, predominantly affecting the liver and lungs.^1^ The WHO estimates that more than 1 million humans are infected globally, with an estimated $3 billion in annual losses due to agricultural and healthcare costs.^2^ The epidemiology, pathogenesis, clinical manifestations, treatment, and prognosis are determined in part by the biology and lifecycle of the *Echinococcus* species involved.

**Figure 1.**
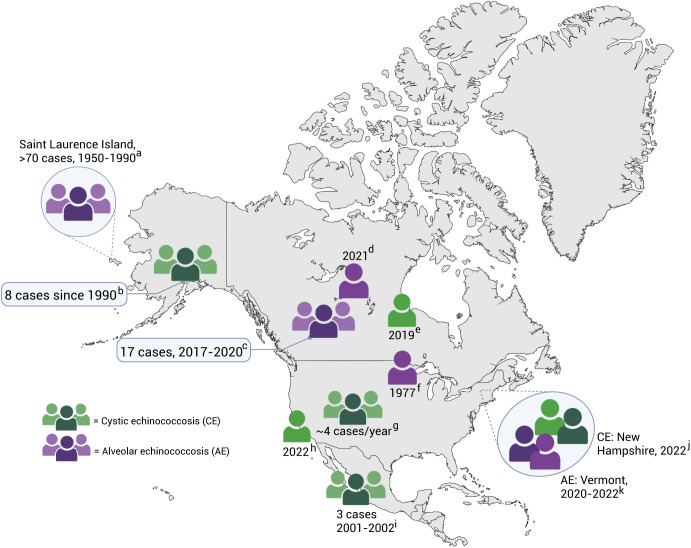
Reports of suspected locally acquired infections with cystic and alveolar echinococcosis, North America, 1977–2022. Each human figure represent one case report, two figures represent two cases, and three grouped figures represent a cluster of ≥3 cases in a region. In the NE US, two reports of each form of echinococcosis are included. Purple = confirmed or suspected alveolar echinococcosis. Green = confirmed or suspected cystic echinococcosis. Sources: ^a^Massolo et al. 2014; ^b^Hueffer et al. 2013; ^c^Houston et al. 2021; ^d^Schurer et al. 2020; ^e^Grocholski et al. 2019; ^f^Yamasaki et al. 2008; ^g^Moro and Schantz 2008; ^h^Passarelli et al. 2022; ^i^Flisser et al. 2015; ^j^AlSalman et al. 2023; and ^k^Polish et al. 2020, 2022.

Although they are considered rare diseases in North America, AE, in particular, is an emerging public health concern in certain geographic localities of the United States and Canada. For example, the recent report of the first US cases of the European haplotype of AE in humans and increasing reports of AE in animals (e.g., coyotes and domestic dogs) highlight the need to conduct surveillance studies in the United States to better understand the geographic distribution, genetic diversity, and factors responsible for its apparent reemergence (Figure 1).^3^^–^^8^ Notably, before the case reported in Vermont in 2020 by Polish et al., AE had not been reported in the contiguous United States since 1977.^9^ Canada has also experienced an increase in reports of newly introduced AE in the Western and Central regions (Saskatchewan, Alberta), in contrast to the steep decline in the previously endemic Holarctic and Northern territories.^10^^–^^13^

Cystic echinococcosis is relatively rare in the United States, with local transmission previously involving a pastoral sheep–dog lifecycle reported in western states (California, Arizona, New Mexico, and Utah), including parts of the Navajo Nation.^14^ In Alaska and Northwest Canada, CE has historically involved domestic dogs and wild ungulates, disproportionately affecting Indigenous communities (American Indian or Alaska Native [AIAN], as reported by the US Census Bureau).^10^ Reports of locally acquired CE in North America remain uncommon. Since 2001, autochthonous CE cases have been reported in California, New Hampshire, and Alaska in the United States,^15^^–^^17^ in a community in Santa Cruz, Mexico,^18^ and in rural Manitoba, Canada.^19^ Compared with historical trends, these reports of locally acquired infections suggest that the geographic range of echinococcosis in North America, particularly AE, is expanding to previously nonendemic regions as reported by the WHO and the World Organization for Animal Health in 2001,^20^ warranting re-evaluation of the baseline disease burden in the United States.

Because echinococcosis is not a consistently reportable disease in North America, the current understanding of its epidemiology in the United States is incomplete, with only a few contemporary US-based reports. Accordingly, significant gaps persist in the current geographic distribution and prevalence of both locally acquired and emigrated cases within the contiguous United States. The aim for the present study is to retrospectively characterize the epidemiological features of echinococcosis diagnoses, both CE and AE, using multicenter deidentified patient data to update the current understanding of this neglected disease’s clinical burden and outcomes in US-based patients.

## MATERIALS AND METHODS

### Global federated research network.

The data used in this study were extracted between January and May 2024 from the TriNetX platform, which provides access to deidentified electronic medical records (diagnoses, procedures, medications, laboratory values, genomic information) from ∼127 million patients reported by 89 healthcare organizations (HCOs) participating in the same research network as the University of Colorado Anschutz Medical Campus. Access was provided by the Health Data Compass of the Research Informatics Office (CU Anschutz, Aurora, CO). Most participating HCOs are large academic medical institutions with inpatient and outpatient facilities. The data represent the entire patient population at the HCO, with an average of 7 years of historical data and a ∼1-month lag time in reporting. TriNetX maps the data to a standard, controlled set of clinical terminologies and transforms it into a proprietary data model. This transformation process includes an extensive data quality assessment that rejects records that do not meet the TriNetX quality standards.

TriNetX complies with the US federal Health Insurance Portability and Accountability Act (HIPAA), certified to the ISO 27001:2013 standard, maintaining an Information Security Management System to meet the requirements of the HIPAA Security Rule. Data are deidentified in accordance with the deidentification standard defined in Section §164.514(a) of the HIPAA Privacy Rule. The process of deidentifying data is attested to through a formal determination as defined in Section §164.514(b)(1) of the HIPAA Privacy Rule. Geographic reporting at the regional level prevents potential reidentification through the localization of patients or HCOs.

### Study design and population.

The index event for echinococcosis was defined as the first recorded diagnosis using *International Classification of Diseases, 10th Revision* (ICD-10) B67 codes, specifying either cystic or alveolar forms of the disease. Patients were categorized into two cohorts based on disease type: CE (*N* = 728; codes B67.0–B67.4) and AE (*N* = 75; codes B67.5–B67.69), including subcodes that specified organ location when applicable. Additional coding of “unspecified echinococcosis” (*N* = 36,260; code B67.9) was noted but excluded from further data collection because of the low specificity of the diagnosis (data not shown). Failing to report sites and species of infection is inconsistent with expert consensus to classify and stage all infections before choosing an appropriate treatment.^21^ To estimate the frequency of diagnosed cases, the average monthly arrival rate of patient encounters with an echinococcosis diagnosis in this HCO network from January 1, 2021 through December 31, 2023 was calculated.

### Baseline population data and outcome measures.

Descriptive findings for patients with AE or CE diagnoses were examined with respect to the following baseline data: regional distribution in the United States, demographics, and clinical baseline characteristics (symptoms, laboratory findings, chronic comorbidities, and diagnostic imaging procedures undertaken). The US regions reflect HCO reporting delineations as defined by TriNetX. Chronic comorbidities were included at any time in the patient record, with laboratory data limited to the most recent observation within 1 year before the index event, except for the “*Echinococcus* sp. IgG immunoassay,” which included the most recent observation from any time point in the patient record.

The primary outcomes assessed post-index included mortality, emergency and critical care encounters, and instances of relevant medical or surgical treatments, limited to records from October 2003 to October 2023, for up to 20 years post-index. Surgeries included select thoracic or abdominal procedures, such as surgery on the liver or lungs and pleura, hepatectomy or lung lobectomy, or cyst aspiration or injection of a hepatic parasitic cyst or abscess, captured using Current Procedural Terminology (CPT) codes. Antiparasitic medication included the anti-cestode drugs albendazole and mebendazole. A Kaplan–Meier analysis in TriNetX was used to estimate survival probability using a daily time interval for up to 20 years after the index event of an echinococcosis diagnosis code. Patients were removed from the survival study (censored) after the last data entry in their records was completed. Proportions receiving treatment and percent survival probabilities at the end of this time window were reported, along with 95% CIs. TriNetX Master Terminology, including several data domains (International Classification of Diseases, Tenth Revision, Clinical Modification (or ICD-10, Clinical Modification, RxNorm, or CPT), was used to define all criteria, procedures, and diagnoses, with codes reported in the text and corresponding tables.

Given the low sample size in the AE cohort, propensity score matching was not performed to balance the cohorts by demographic features. Therefore, formal hypothesis testing was not conducted between cohorts because of confounding patient variables. The programs RStudio (v.2023.03.0 + 386; Posit Software PBC, Boston, MA), GraphPad Prism 9 (GraphPad Software, San Diego, CA), BioRender.com, and Microsoft Excel (Microsoft 365 v.16.84, Microsoft Corp., Redmond, CA) were used with TriNetX output summary data for further analysis and data visualization.

## RESULTS

**Figure 2.**
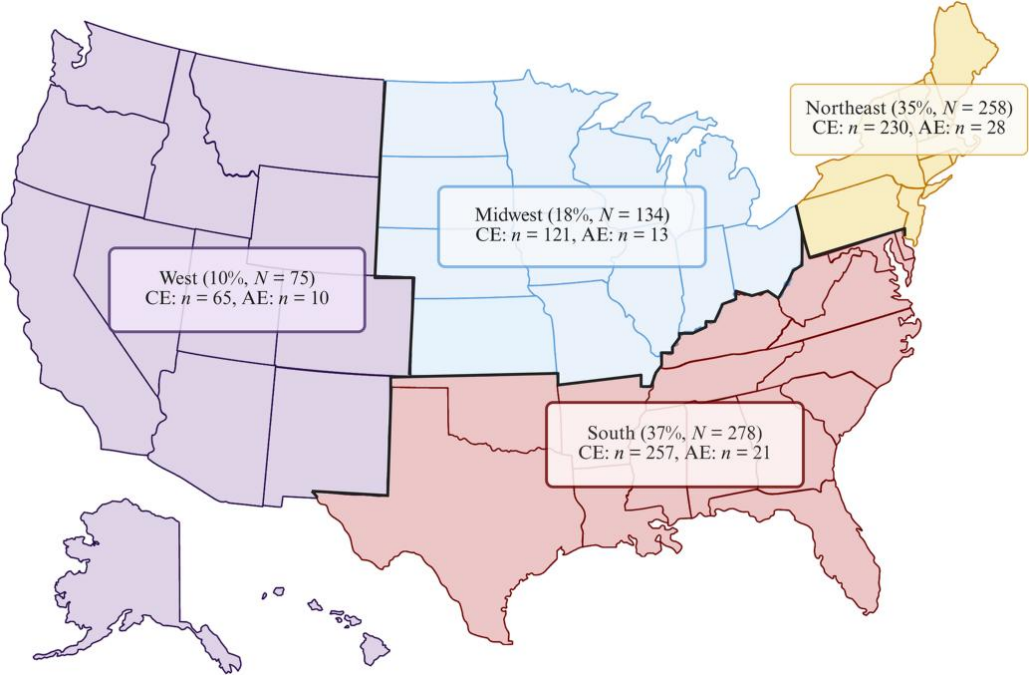
Regional distribution of echinococcosis in the United States by species group. Distribution of deidentified US echinococcosis diagnosis codes (ICD10: B67 tree) by reporting healthcare organizations in TriNetX. CE = cystic echinococcosis caused by *Echinococcus granulosus sensu lato* (*N* = 728). AE = alveolar echinococcosis caused by *E. multilocularis* (*N* = 75). Regional borders and proportion of echinococcosis diagnosis group by US region as reported by TriNetX. There were 55 CE and 3 AE patients excluded with extra-US or unknown locations. The South (red) and Northeast (yellow) comprised the largest share of AE and CE codes, comprising 72% of all cases.

Across 68 of 89 HCOs contributing to the TriNetX research network, 36,919 patients with any B67 diagnosis code for echinococcosis were identified, out of ∼126.6 million total patient records. These records spanned all available years, with 95% recorded between 2004 and 2022. Less than 1% of patients were reported from an unknown or non-US location. More than 98% of echinococcosis diagnoses were coded as “unspecified echinococcosis” (B67.8–B67.9) and were excluded from further analysis because of the lack of diagnostic specificity. After excluding these cases, 75 patients with a diagnosis code for AE (B67.5–B67.7) and 728 with a diagnosis code for CE (B67.0–B67.4) were analyzed in this US-based HCO network, as of May 22, 2024. For post-index outcome analyses, patient records were restricted to the period spanning from October 2003 to October 2023, resulting in the exclusion of 15 AE and 177 CE patients. Geographically, the South and Northeast US regions accounted for most cases of AE and CE, representing more than two-thirds of the total caseload. The South had the greatest number of CE cases (*n* = 257), whereas the Northeast reported the most AE cases (*n* = 28) in the United States (Figure 2).

### Demographics.

The average ages of patients with CE and AE were 53 and 61 years, respectively; 55% and 67% were female, respectively. Representation of racial and ethnic groups differed by diagnosis type, with the majority being white (52% CE; 61% AE) and not Hispanic or Latino (54% CE; 49% AE). No AIAN patients were reported in either cohort from this network. More than one-quarter of patients were listed as of unknown race (Table 1).

**Table 1:** Patient demographics associated with cystic and alveolar echinococcosis, US database (1997–2024)

Population and Demographics	Cystic Echinococcosis	Alveolar Echinococcosis
Healthcare organizations (out of 89)	65	27
Total patients	728	75
Rate of arrival (monthly average/2021–2023)	3.2	0.4
Time window for 95% of patient data	2002–2023	1997–2024
Average age, years (SD)	53 (23)	61 (21)
Sex (% of total)		
Female	487 (67%)	41 (55%)
Male	233 (32%)	31 (41%)
Unknown	8 (1%)	3 (4%)
Ethnicity (% of total)		
Not Hispanic or Latino	395 (54%)	37 (49%)
Unknown	263 (36%)	32 (43%)
Latino or Hispanic	70 (10%)	6 (8%)
Race (% of total)		
White	377 (52%)	46 (61%)
Unknown	192 (26%)	22 (29%)
Black or African American	96 (13%)	1 (1%)
Other	38 (5%)	5 (7%)
Asian	23 (3%)	1 (1%)
Native Hawaiian or Pacific Islander	2 (<1%)	0
American Indian or Alaska Native	0	0

### Clinical manifestations and diagnostics.

Few clinical signs and symptoms were noted in the 12 months before an echinococcosis diagnosis, with 5% of patients in each group having experienced abdominal or pelvic pain. However, 31% experienced chronic malaise or fatigue, and 6–7% experienced ascites at any time point. Different diagnostic imaging procedures of the chest and abdomen were performed for 11–36% of patients across both groups. The most common imaging study was a radiologic examination of the chest, followed by computed tomography of the thorax, abdomen, or pelvis, and then abdominal ultrasound (Table 2).

**Table 2:** Clinical signs and diagnostic imaging procedures

Signs/Symptoms (12 months prior)	CE	AE	ICD-10	CPT
Abdominal/pelvic pain	40 (5%)	4 (5%)	R10	–
Nausea/vomiting	25 (3%)	2 (3%)	R11	–
Pain in throat/chest	21 (3%)	1 (1%)	R07	–
Cough	25 (3%)	1 (1%)	R05	–
Headache	18 (2%)	2 (3%)	R51	–
Fever of unknown origin	13 (2%)	1 (1%)	R50	–
Hepatomegaly/splenomegaly	12 (2%)	0	R16	–
Chronic symptoms (anytime)				
Malaise/fatigue	228 (31%)	23 (31%)	R53	–
Ascites	45 (6%)	5 (7%)	R18	–
Cachexia	21 (3%)	2 (3%)	R64	–
Diagnostic imaging (anytime)				
Diagnostic radiology of chest	263 (36%)	25 (33%)	–	1031050
CT of the abdomen and pelvis	233 (32%)	24 (32%)	–	1020544
Abdominal ultrasonography	214 (29%)	22 (29%)	–	1010775
CT of thorax	201 (28%)	26 (35%)	–	1036223
Radiologic exam abdomen	162 (22%)	11 (15%)	–	1031051
MRI abdomen	78 (11%)	11 (15%)	–	1010531

AE = alveolar echinococcosis; CE = cystic echinococcosis; CPT = Current Procedural Terminology; CT = computed tomography; ICD-10 = *International Classification of Diseases, 10th Revision*; MRI = magnetic resonance imaging. Procedures included full examinations, excluding cardiac or reproductive-specific imaging studies. Symptoms and procedures were captured using ICD-10 or CPT codes reported in patient records, relative to an echinococcosis diagnosis in a US-based network.

**Figure 3.**
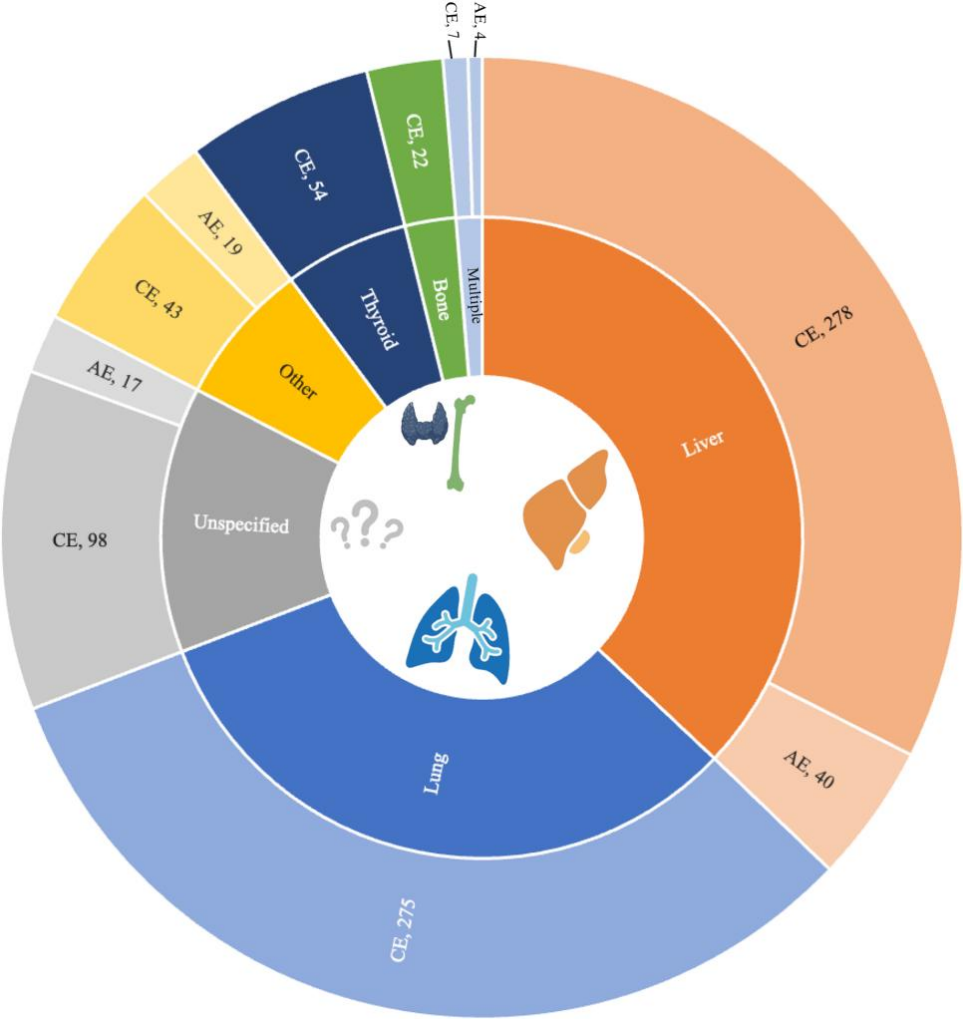
Reported organ distribution for echinococcosis-related codes. Affected organs are based on sub-diagnosis codes of echinococcosis (ICD10 B67 tree) as reported by healthcare organizations in the US. The inner ring represents the total pooled proportion by reporting category, with the outer ring separated by the number of reports in either alveolar echinococcosis (AE, *N* = 75) or cystic echinococcosis (CE, *N* = 728). Patients may have more than one organ location code reported. Most AE and CE cases were reported in the liver (orange) and the lung (blue, CE only).

According to CE and AE sub-codes, the most commonly involved organs were the liver (36% for CE and 50% for AE), followed by the lungs (36%; CE only). Patients with CE had the greatest number of organ site involvement, including 54 (7%) thyroid infections and 22 (3%) bone infections. However, nearly half of AE cases (49%) lacked a specified organ site. The least frequently reported category for both CE and AE was “multiple sites” (Figure 3).

For comorbidities and other underlying potential risk factors for disease complications or immune suppression that may affect prognosis, >40% of patients had primary hypertension, 20–25% had type 2 diabetes mellitus, and 10–15% had heart failure or chronic kidney disease. Fewer than 7% had codes related to socioeconomic or psychosocial health hazards (social determinants of health), transplant history, or HIV. Additional diagnoses of the lung or liver revealed that 21–33% of patients had “other, specified disease of the liver” before an echinococcosis diagnosis, 21% of AE patients reported chronic passive congestion of the liver, and 19% of CE patients had asthma. Beyond “unspecified liver disease,” all other liver conditions considered (chronic viral hepatitis, abscess, fibrosis, and cirrhosis of the liver) affected less than 10% of patients. A total of 9–11% of patients had chronic obstructive pulmonary disease, and very rarely, patients had tuberculosis (1–2%). Only 5% or fewer patients reported malignant neoplasia of the digestive organs, liver, respiratory or intrathoracic organs, or bronchus or lung (Table 3).

**Table 3:** Co-occurring health conditions and complication risk factors in US echinococcosis patients

Chronic Comorbidities and Risk Factors	CE	AE	ICD-10
Primary hypertension	289 (41%)	34 (45%)	I10
Type 2 diabetes mellitus	146 (20%)	19 (25%)	E11
Chronic kidney disease	80 (11%)	11 (15%)	N18
Heart failure	76 (10%)	8 (11%)	I50
Social determinants of health hazards	52 (7%)	5 (7%)	Z55–Z65
Transplantation status	29 (4%)	3 (4%)	Z94
HIV disease	16 (2%)	0	B20
Liver conditions			
Other specified diseases of the liver	150 (21%)	25 (33%)	K76.89
Chronic passive congestion of the liver	62 (9%)	16 (21%)	K76.1
Liver disease, unspecified	58 (8%)	8 (11%)	K76.9
Abscess of liver	30 (4%)	5 (7%)	K75.0
Fibrosis and cirrhosis of the liver	32 (4%)	3 (4%)	K74
Chronic viral hepatitis	21 (3%)	0	B18
Liver transplant status	11 (2%)	0	Z94.4
Pulmonary conditions			
Asthma	135 (19%)	4 (5%)	J45
Chronic obstructive pulmonary disease	69 (9%)	8 (11%)	J44
Tuberculosis	11 (2%)	1 (1%)	A15–A19
Malignancies			
Digestive organ neoplasms	35 (5%)	3 (4%)	C15–C26
Respiratory/intrathoracic neoplasms	24 (3%)	3 (4%)	C30–C39
Bronchus/lung malignancy	18 (2%)	3 (4%)	C34
Liver malignancy	13 (2%)	0	C22

AE = alveolar echinococcosis; CE = cystic echinococcosis; ICD-10 = *International Classification of Diseases, 10th Revision*. All noted ICD-10 codes were included from any time point in the patient record.

The values for routine laboratory blood results in the year before the index diagnosis largely fell within normal limits (according to American Board of Internal Medicine Laboratory test reference ranges, January 2024). The 95% CIs included values above the upper limits for total white blood cell (leukocyte) counts and liver enzymes (Alanine transaminase (ALT), Aspartate transaminase (AST), and Alkaline phosphatase (ALP)), as well as below the lower limits for hematocrit and albumin. The averaged *Echinococcus* sp. IgG immunoassay (Logical Observation Identifiers Names and Codes 9656.0) value was above the positive threshold for this test code (>1.009), with CE patients exhibiting higher test positivity (74%) and higher antibody titers (Table 4).

**Table 4:** Most recent reported clinical laboratory values and *Echinococcus* spp. antibody titers

Laboratory Values (units)	CE, Average ± SD (*n*)	AE, Average ± SD (*n*)
Leukocytes (10^3^/*µ*L)	7.55 ± 3.03 (100)N↑	6.72 ± 2.55 (10)N
Eosinophils (% of 100 leukocytes)	2.69 ± 2.81 (103)N	3.55 ± 1.94 (11)N
Hematocrit [%]	39.4 ± 5.44 (119)N↓	37.2 ± 8.67 (12)N↓
Alanine aminotransferase (U/L)	28.3 ± 56.4 (116)N↑	25 ± 20.9 (12)N↑
Aspartate transaminase (U/L)	26.3 ± 17.4 (107)N↑	29.2 ± 15.9 (12)N↑
Alkaline phosphatase (U/L)	92.5 ± 52.8 (107)N	83.4 ± 67.1 (12)N↑
Bilirubin total (mg/dL serum)	0.593 ± 0.396 (106)N	0.536 ± 0.347 (11)N
Albumin (g/dL serum)	4.06 ± 0.705 (107) N↓	3.78 ± 0.82 (11)N↓
Total protein (g/dL serum)	7.08 ± 0.678 (88)N	7.25 ± 0.864 (11)N
* Echinococcus* spp. IgG (arb’U/mL)		
Avg. ± SD (*n*)	7.5 ± 8.96 (23)↑	1.48 ± 1.01 (6)↑
Above positive threshold/total tested	17/23 (74%)	4/6 (67%)
Range of values	0–33	0.7–3.5

For 95% CIs, N = falls within normal limits, N↑ = normal to above upper limits, N↓ = normal to below lower limits, ↑ = above upper limits. Interpretation cutoffs for IgG immunoassay: <0.900 = negative, 0.900–1.1 = equivocal, ≥1.2 = positive (Logical Observation Identifiers Names and Codes 9656-0). This table includes the most recent patient observation within 12 months before index diagnosis reported for all laboratory values, except for *Echinococcus* sp. IgG immunoassay, which revealed the most recent observation from any time in the patient record.

**Figure 4.**
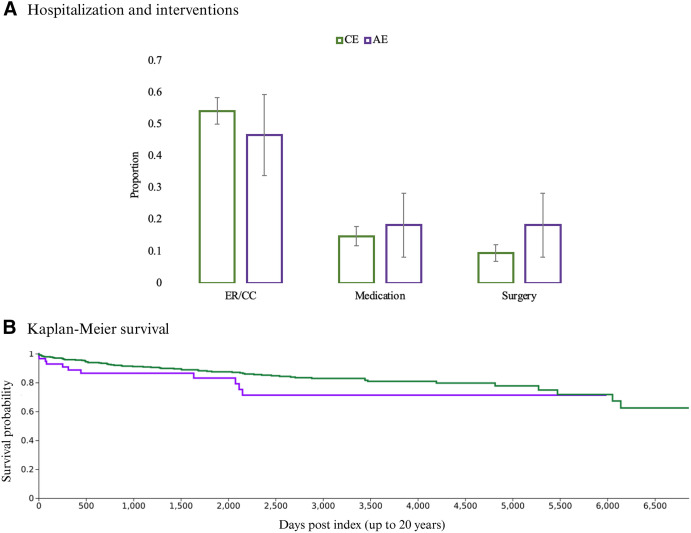
Hospitalization, interventions, and survival probability for patients with echinococcosis. (**A**) Proportion of patients with cystic echinococcosis (CE, green) or alveolar echinococcosis (AE, purple) experiencing relevant treatments or hospital services over 20 year follow-up. ER/CC = emergency or critical care encounters, medication = albendazole and/or mebendazole, surgery = thoracic or abdominal procedures (described in text). (**B**) Average percent probability of survival calculated by TriNetX for up to 20 years (last patient record 16 years post-index for AE, 20 years for CE) following echinococcosis diagnosis. Patients were removed (censored) after the last data entry in their medical records.

Patients coded for either AE and CE had similarly high proportions of emergency or critical care hospital encounters (CPT codes 1013711 and 1013729). An estimated 55% (95% CI: 50–58%) of CE patients and 46% (95% CI: 33–59%) of AE patients experienced such encounters. Albendazole or mebendazole (RxNorm codes 430 and 2456) were administered to 15% (95% CI: 12–18%) of patients with CE and 18% (95% CI: 8–28%) of patients with AE. When more than one treatment with an anticestode medication was reported, the median number of retreatment instances was three total treatments for CE patients and five total treatments for AE patients. Any thoracic or abdominal surgical procedures (CPT codes 1004142, 1007795, 1007845, and 1005955), including laparotomy with injection or aspiration of parasitic (amoebal or echinococcal) cysts (CPT 47015; recommended for CE only), occurred in a total of 10% (95% CI: 7–12%) of patients with CE and 18% (95% CI: 8–28%) of patients with AE (Figure 4A). The cumulative survival probability at 20 years post-index was 71% (95% CI: 60–82%) for patients with AE and 62% (95% CI: 58–66%) for patients with CE, with 11 AE and 69 CE deaths recorded. Patients with AE experienced earlier mortality when compared with those with CE, with all 11 recorded deaths occurring within the first 6 years (Figure 4B).

## DISCUSSION

A comprehensive and contemporary summary of patients diagnosed with echinococcosis in the United States is presented, including ∼800 cases from an analysis of 127 million deidentified patient records from the past three decades. Data from 2021 to 2023 suggested an average of four cases per month presented for care across these 68 HCOs. Although they constitute only a small fraction of the population, the treatment of CE and AE patients represents a significant clinical burden. Although locally acquired cases remain rare, the geographic distribution of these cases has changed when compared with recent reviews of North America.^3^^,^^10^^,^^20^^,^^22^^,^^23^ At present, verification of the clinical criteria used for these diagnoses and identification of the infections’ geographic origin are not possible. When true infections are represented, the study findings suggest that many patients diagnosed with echinococcosis in the United States receive insufficient treatment and few staging or classification diagnostics, based on the limited deidentified aggregate CPT and ICD-10 codes. Distinct differences persisted between patients coded as CE versus AE.

Cystic echinococcosis, which involves species in the *E. granulosus sensu lato* complex, has a near-cosmopolitan distribution.^24^ In humans, unilocular larval cysts primarily affect the liver or the lung, remaining asymptomatic for many years in the absence of complications.^25^ Although responsible for most of the global burden, CE is a rare entity in the United States, with only a handful of recent case reports (Figure 1).^15^^,^^16^^,^^26^ Contrary to this notion, the study database contained 728 diagnoses of CE, including 551 cases recorded between 2003 and 2023. Historically, CE disproportionately impacted Indigenous communities in Alaska, Northern Canada, and the Western United States.^14^^,^^27^ In contrast, no cases of echinococcosis were found among individuals identifying as AIAN in the present study (Table 1). In addition, the Western United States reported the fewest cases of CE, with the largest proportion reported in the South (Figure 2). For example, the Texas Department of State Health Services has included echinococcosis as a reportable zoonotic disease since 2016, with 22 provisional human cases as of May 31, 2025, the majority of which are assumed to have been acquired in other endemic countries (https://www.dshs.texas.gov/notifiable-conditions/zoonosis-control/zoonotic-disease-cases/human-cases-2020-2024). Further studies are needed to ascertain the potential sources of CE in the United States, including the distribution of different genotypes among animal populations.^22^ Moreover, the authors of future investigations should build upon the recent findings of CE in slaughterhouses and canines in the United States, as animal surveillance is essential for monitoring local public health risks associated with CE.^28^^,^^29^

Alveolar echinococcosis, which is caused by *E. multilocularis*, is a more severe and aggressive disease. Complex, infiltrative, and multilocular cysts that predominantly affect the liver behave like a malignancy, with “metastatic-like spread” to distant sites, contributing to a poor prognosis.^30^ It is noteworthy that 60 patients in the United States were coded with AE over the past two decades. This figure is particularly important given that the more virulent and recently introduced European haplotypes of *E. multilocularis* have been spreading across Canada since 2012, initially in animal populations and later in humans.^12^^,^^13^^,^^31^ Alveolar echinococcosis has been recently reported in coyotes in New York, a dog in Virginia, and two humans in Vermont, who harbored genetically similar tapeworms to those found in local foxes (Figure 1).^32^^–^^34^ Potentially consistent with these locally acquired reports, most of the cases of AE in the study database were reported in the Northeastern United States (Figure 2). This finding underscores the need to conduct genetic characterization of the parasites in both animals and humans in suspected cases of locally acquired AE in the United States.

The source of these infections is an important unanswered question. The United States is home to a large number of foreign-born residents, comprising 14.3% of the total population as of 2023 (US Census Bureau, 2023 American Community Survey, 2024 Current Population Survey, and 2000 decennial census). The Centers for Disease Control and Prevention currently states that most cases of echinococcosis described in the United States were acquired from endemic foreign countries.^35^ A review of US mortality records from 1990 to 2007 revealed 41 echinococcosis-attributed deaths in public records, compared with the 80 deaths in this database from 2003 to 2023. Of those 41 cases, 16 (39%) were US-born, leaving the possibility of local exposure unresolved.^23^ Similar to the limitations of the present study, additional travel history could not be determined. The true presence, extent, prevalence, and current transmission dynamics of locally acquired echinococcosis in the United States remain largely unknown.^3^^,^^22^^,^^27^^,^^36^ Regardless of known source attribution, understanding the clinical management of these cases and their impact on the US health system remains valuable.

In the present study, nonspecific clinical manifestations, such as chronic malaise or fatigue, were observed in up to one-third of patients, with a slight increase in leukocytosis and elevated liver enzymes (Tables 2 and 4). Imaging studies, which are pivotal in the diagnosis of echinococcosis, were infrequently performed in the cohort (Table 4).^21^ As an adjunctive tool to support the diagnosis, antibody testing was performed less frequently, with negative or equivocal results in 6/23 (26%) and 2/6 (33%) of all CE and AE patients tested, respectively. The remarkably high use of nonspecific codes for echinococcosis (B67.9; >36,000 unspecified species, unspecified site) also indicates higher uncertainty in the differential diagnosis or incomplete details in the medical records. Regarding treatment and outcomes, high rates of probable observation without medical or surgical intervention were found for both CE and AE (Figure 4). Importantly, a conservative wait-and-see approach can be acceptable for inactive CE cysts but is not appropriate for AE.^37^ Independent of the initial treatment choice, long-term monitoring of at least 10 years is critical because inactive cysts can revert to an active stage with a 6.5% post-intervention relapse rate.^38^ These findings underscore the potential underappreciation of echinococcosis in the appropriate clinical setting and the unfamiliarity of healthcare professionals with treatment guidelines, which, for CE, are dependent on classification via imaging studies. Appropriate treatment is paramount to improving patient outcomes and survival.

The long-term morbidity and mortality impact of echinococcosis in the present study is noteworthy (Figure 4). Approximately half of the patients required emergency room or critical care encounters during the 20-year follow-up period. According to the 2021 US Social Security Actuarial Life Table (www.ssa.gov/oact/STATS/table4c6.html), the average patient with echinococcosis in the United States has a 14% higher risk of mortality over 20 years when compared with an age-matched general US adult female population. Comparing rates of chronic comorbidities for these patients to six published case series in other countries,^30^^,^^39^^–^^43^ the study dataset revealed higher prevalences of type 2 diabetes (12.1% versus 20–25% in US patients), chronic kidney disease (2.8% versus 11–15%), and heart failure (7.2% versus 10–11%) in US-based patients (Table 2). Immunosuppression is an understudied risk factor for the development and progression to severe disease in patients with AE, with immunosenescence being a potential factor for the increased mortality risk associated with older age.^10^^,^^23^

The retrospective nature of this cohort is susceptible to selection bias. This dataset is limited to the 89 large healthcare institutions participating in this research network. It may not represent other types of health institutions, such as smaller rural practices or Indian Health Board centers, leading to underrepresentation of certain groups. A major limitation of the present study was the inability to distinguish between locally acquired and imported infections. Additionally, medical record coding inaccuracies and incompleteness have been documented as a significant issue for research, with one audit revealing a 10% error rate in coding at a tertiary care center.^44^^,^^45^ Despite these limitations, the authors are confident that these diagnostic billing codes were accurately transmitted from true electronic medical record data entries into this database.

## CONCLUSION

The study data serve as an updated baseline for ongoing surveillance of patients with echinococcosis presenting for care in the United States, a key public health metric in light of recent reports of locally acquired echinococcoses in North America. The development of a prospective multiregional online registry of patients and animals with CE and AE, including a biorepository similar to the decade-old European Register of CE, should be prioritized in North America. This information is crucial for the development of effective, efficient, and sustainable preventive and control measures, which align with the WHO’s 2030 echinococcosis global control goals.^46^ Increasing awareness of the epidemiological, clinical, and therapeutic aspects of the disease among healthcare professionals is equally important.

## Data Availability

Data availability: The corresponding author had full access to the study data through the University of Colorado Anschutz Health Data Compass and was responsible for submitting the manuscript for publication. The datasets generated and analyzed in the current study are available from TrinetX for institutions that have a subscription to the platform.

## References

[1] McManus DP Gray DJ Zhang W Yang Y, 2012. Diagnosis, treatment, and management of echinococcosis. BMJ 344: e3866. 22689886 10.1136/bmj.e3866

[2] World Health Organization, 2021. *Fact Sheets: Echinococcosis*. Available at: https://www.who.int/news-room/fact-sheets/detail/echinococcosis. Accessed March 17, 2023.

[3] Massolo A Liccioli S Budke C Klein C, 2014. *Echinococcus multilocularis* in North America: The great unknown. Parasite 21: 73. 25531581 10.1051/parasite/2014069 PMC4273702

[4] Garrett K Brown J Grunert RKA Hunte J Ruder MG Van Why K Yabsley MJ Cleveland CA, 2023. *Echinococcus* species infections among wild canids in Pennsylvania, USA. *J Wildl Dis* 59: 332–336. 37036486 10.7589/JWD-D-22-00042

[5] Kuroki K Morishima Y Dorr L Cook CR, 2022. Alveolar echinococcosis in a dog in Missouri, USA. *J Vet Diagn Invest* 34: 746–751. 35678137 10.1177/10406387221104754 PMC9266511

[6] Schurer JM Bouchard E Bryant A Revell S Chavis G Lichtenwalner A Jenkins EJ, 2018. *Echinococcus* in wild canids in Québec (Canada) and Maine (USA). PLoS Negl Trop Dis 12: e0006712. 30125277 10.1371/journal.pntd.0006712 PMC6117095

[7] Polish LB Pritt B Barth TFE Gottstein B O’Connell EM Gibson PC, 2021. First European haplotype of *Echinococcus multilocularis* identified in the United States: An emerging disease? Clin Infect Dis 72: 1117–1123. 32198510 10.1093/cid/ciaa245 PMC8028098

[8] Kuroki K Morishima Y Neil J Beernstsen BT Matsumoto J Stich RW, 2020. Intestinal echinococcosis in a dog from Missouri. *J Am Vet Med Assoc* 256: 1041–1046. 32301665 10.2460/javma.256.9.1041

[9] Yamasaki H Nakao M Nakaya K Schantz PM Ito A, 2008. Genetic analysis of *Echinococcus multilocularis* originating from a patient with alveolar echinococcosis occurring in Minnesota in 1977. *Am J Trop Med Hyg* 79: 245–247. 18689631

[10] Davidson RK Lavikainen A Konyaev S Schurer J Miller AL Oksanen A Skirnisson K Jenkins E, 2016. *Echinococcus* across the north: Current knowledge, future challenges. Food Waterborne Parasitol 4: 39–53.

[11] Houston S, , 2021. Epidemiological and clinical characteristics of alveolar echinococcosis: An emerging infectious disease in Alberta, Canada. *Am J Trop Med Hyg* 104: 1863–1869. 33755579 10.4269/ajtmh.20-1577 PMC8103444

[12] Schurer JM Tsybina P Gesy KM Kolapo TU Skinner S Hill JE Jenkins EJ, 2021. Molecular evidence for local acquisition of human alveolar echinococcosis in Saskatchewan, Canada. *J Infect Dis* 223: 1015–1018. 32766836 10.1093/infdis/jiaa473

[13] Santa MA Umhang G Klein C Grant DM Ruckstuhl KE Musiani M Gilleard JS Massolo A, 2023. It’s a small world for parasites: Evidence supporting the North American invasion of European *Echinococcus multilocularis*. *Proc Biol Sci* 290: 20230128. 36883278 10.1098/rspb.2023.0128 PMC9993045

[14] Moro P Schantz PM, 2009. Echinococcosis: A review. *Int J Infect Dis* 13: 125–133. 18938096 10.1016/j.ijid.2008.03.037

[15] Passarelli P Ramchandar N Naheedy J Kling K Choi L Pong A, 2022. An 8-year-old California girl with asymptomatic hepatic cysts. *Pediatr Infect Dis J* 41: e295–e296. 35421052 10.1097/INF.0000000000003539

[16] AlSalman A Mathewson A Martin IW Mahatanan R Talbot EA, 2023. Cystic echinococcosis in Northern New Hampshire, USA. *Emerg Infect Dis* 29: 1057–1058. 37044131 10.3201/eid2905.221828 PMC10124641

[17] Hueffer K Parkinson AJ Gerlach R Berner J, 2013. Zoonotic infections in Alaska: Disease prevalence, potential impact of climate change and recommended actions for earlier disease detection, research, prevention and control. *Int J Circumpolar Health* 2013: 72. 10.3402/ijch.v72i0.19562 PMC3568173 23399790

[18] Flisser A Maravilla P Mata-Miranda P Martinez-Hernandez F, 2015. Echinococcosis in Mexico—A story worth sharing. Rodriguez-Morales AJ, ed. Current Topics in Echinococcosis. Rijeka, Croatia: IntechOpen, 31–53.

[19] Grocholski S Agabawi S Kadkhoda K Hammond G, 2019. *Echinococcus granulosus* hydatid cyst in rural Manitoba, Canada: Case report and review of the literature. IDCases 18: e00632. 31700800 10.1016/j.idcr.2019.e00632 PMC6831802

[20] World Health Organization, 2001. *WHO/OIE Manual on Echinococcosis in Humans and Animals*. Available at: https://www.who.int/publications/i/item/929044522X. Accessed January 13, 2026.

[21] Brunetti E Kern P Vuitton DA, 2010. Expert consensus for the diagnosis and treatment of cystic and alveolar echinococcosis in humans. Acta Trop 114: 1–16. 19931502 10.1016/j.actatropica.2009.11.001

[22] Cerda JR Buttke DE Ballweber LR, 2018. *Echinococcus* spp. tapeworms in North America. *Emerg Infect Dis* 24: 230–235. 29350139 10.3201/eid2402.161126 PMC5782903

[23] Bristow BN Lee S Shafir S Sorvillo F, 2012. Human echinococcosis mortality in the United States, 1990–2007. PLoS Negl Trop Dis 6: e1524. 22347516 10.1371/journal.pntd.0001524 PMC3274497

[24] Wen H Vuitton L Tuxun T Li J Vuitton DA Zhang W McManus DP, 2019. Echinococcosis: Advances in the 21st century. *Clin Microbiol Rev* 32: e00075-18. 30760475 10.1128/CMR.00075-18 PMC6431127

[25] Govindasamy A Bhattarai PR John J, 2023. Liver cystic echinococcosis: A parasitic review. *Ther Adv Infect Dis* 10: 20499361231171478. 37197609 10.1177/20499361231171478 PMC10184195

[26] Manterola C Totomoch-Serra A Rojas C Riffo-Campos ÁL García-Méndez N, 2022. *Echinococcus granulosus sensu lato* genotypes in different hosts worldwide: A systematic review. Acta Parasitol 67: 161–185. 34264444 10.1007/s11686-021-00439-8

[27] Hotez PJ, 2011. Neglected infections of poverty in the United States of America. Institute of Medicine (US) Forum on Microbial Threats, ed. The Causes and Impacts of Neglected Tropical and Zoonotic Diseases: Opportunities for Integrated Intervention Strategies. Washingtion, DC: National Academies Press, 237–263. 21977543

[28] Jesudoss Chelladurai JRJ Quintana TA Johnson WL Schmidt C Righter D Howey E, 2024. Cystic echinococcosis in cattle and sheep caused by *Echinococcus granulosus sensu stricto* genotypes G1 and G3 in the USA. Parasit Vectors 17: 128. 38486339 10.1186/s13071-024-06192-x PMC10938798

[29] Bernstein LA Shaffer C Walz E Moore S Sparks A Stone S Roerick T Larsen PA Wolf TM, 2021. Exploring risk for echinococcosis spillover in northern Minnesota tribal communities. EcoHealth 18: 169–181. 34508275 10.1007/s10393-021-01547-7

[30] Grüner B Kern P Mayer B Gräter T Hillenbrand A Barth TEF Muche R Henne-Bruns D Kratzer W Kern P, 2017. Comprehensive diagnosis and treatment of alveolar echinococcosis: A single-center, long-term observational study of 312 patients in Germany. GMS Infect Dis 5: Doc01. 30671323 10.3205/id000027 PMC6301735

[31] Massolo A, , 2019. European *Echinococcus multilocularis* identified in patients in Canada. N Engl J Med 381: 384–385. 31340100 10.1056/NEJMc1814975

[32] Polish LB, , 2022. European haplotype of *Echinococcus multilocularis* in the United States. *N Engl J Med* 387: 1902–1904. 36383717 10.1056/NEJMc2210000 PMC10072850

[33] Conlon CL Schuler KL Lejeune M Whipps CM, 2023. Novel report of the European variant of *Echinococcus multilocularis* in coyotes (*Canis latrans*) in New York state. J Parasitol 109: 357–361. 37527278 10.1645/22-104 PMC10658865

[34] Zajac A Fairman D McGee E Wells B Peregrine A Jenkins E LeRoith T St John B, 2020. Alveolar echinococcosis in a dog in the eastern United States. *J Vet Diagn Invest* 32: 742–746. 32715926 10.1177/1040638720943842 PMC7488973

[35] Centers for Disease Control and Prevention, 2019. *Echinococcosis. Division of Parasitic Diseases and Malaria*. Available at: https://www.cdc.gov/dpdx/echinococcosis/. Accessed April 6, 2023.

[36] Torgerson PR Robertson LJ Enemark HL Foehr J van der Giessen JWB Kapel CMO Klun I Trevisan C, 2020. Source attribution of human echinococcosis: A systematic review and meta-analysis. PLoS Negl Trop Dis 14: e0008382. 32569309 10.1371/journal.pntd.0008382 PMC7332091

[37] Lissandrin R Tamarozzi F Mariconti M Manciulli T Burnetti E Vola A, 2018. Watch and wait approach for inactive echinococcal cyst of the liver: An update. *Am J Trop Med Hyg* 99: 375–379. 29869600 10.4269/ajtmh.18-0164 PMC6090333

[38] Kern P da Silva AM Akhan O Mullhaupt B Vizcaychipi KA Budke C Vuitton DA, 2017. The echinococcoses: Diagnosis, clinical management and burden of disease. *Adv Parasitol* 96: 259–369. 28212790 10.1016/bs.apar.2016.09.006

[39] Lopez-Bernus A Belhassen-García M Alonso-Sardón M Carpio-Perez A Velasco-Tirado V Romero-Alegria A Muro A Cordero-Sánchez M Pardo-Lledias J, 2015. Surveillance of human echinococcosis in Castilla-Leon (Spain) between 2000–2012. PLoS Negl Trop Dis 9: e0004154. 26484764 10.1371/journal.pntd.0004154 PMC4618931

[40] Li S, , 2020. Clinical features, radiological characteristics, and outcomes of patients with intracranial alveolar echinococcosis: A case series from Tibetan areas of Sichuan Province, China. *Front Neurol* 11: 537565. 33519658 10.3389/fneur.2020.537565 PMC7843382

[41] Manterola C Claros N Grande L, 2023. Postoperative complications and recurrence of abdominal echinococcosis rupture: Case series with follow-up. Indian J Surg 85: 771–777.

[42] Manterola C, 2021. Extra-visceral retroperitoneal echinococcosis. Case series with follow-up. *Int J Morphol* 39: 386–389.

[43] Collado-Aliaga J Romero-Alegría A Alonso-Sardón M Muro A López-Bernus A Velasco-Tirado V Bellido JLM Pardo-Lledias J Belhassen-García M, 2019. Complications associated with initial clinical presentation of cystic echinococcosis: A 20-year cohort analysis. *Am J Trop Med Hyg* 101: 628–635. 31359859 10.4269/ajtmh.19-0019 PMC6726966

[44] Farhan J Al-Jummaa S Al-Rajhi A Al-Rayes H Al-Nasser A, 2005. Documentation and coding of medical records in a tertiary care center: A pilot study. *Ann Saudi Med* 25: 46–49. 15822494 10.5144/0256-4947.2005.46 PMC6150569

[45] Alonso V Santos JV Pinto M Ferreira J Lema I Lopes F Freitas A, 2020. Health records as the basis of clinical coding: Is the quality adequate? A qualitative study of medical coders’ perceptions. Health Inf Manag 49: 28–37. 30744403 10.1177/1833358319826351

[46] World Health Organization, 2020. *Ending the Neglect to Attain the Sustainable Development Goals: A Framework for Monitoring and Evaluating Progress of the Road Map for Neglected Tropical Diseases 2021–2030*. Available at: https://www.who.int/publications/i/item/9789240010352. Accessed January 23, 2026.

